# Rooting behaviour and soil properties in different bamboo species of Western Himalayan Foothills, India

**DOI:** 10.1038/s41598-020-61418-z

**Published:** 2020-03-18

**Authors:** R. Kaushal, Indra Singh, S. D. Thapliyal, A. K. Gupta, D. Mandal, J. M. S. Tomar, Ambrish Kumar, N. M. Alam, D. Kadam, D. V. Singh, H. Mehta, Pradeep Dogra, P. R. Ojasvi, S. Reza, J. Durai

**Affiliations:** 1 ICAR-Indian Institute of Soil and Water Conservation, 218, Kaulagarh Road, Dehradun, 248 195 India; 2 International Bamboo and Rattan Organization, Addis Ababa, Ethiopia

**Keywords:** Forestry, Plant ecology

## Abstract

Due to extensive root system, connected rhizome bamboos are considered suitable for improving soil properties within a short period, though most of the claims are anecdotal and need to be supported with quantified data. The study evaluates seven bamboo species viz., *Bambusa balcooa, Bambusa bambos, Bambusa vulgaris, Bambusa nutans, Dendrocalamus hamiltonii, Dendrocalamus stocksii* and *Dendrocalamus strictus* for their rooting pattern and impact on soil health properties. Coarse and fine root intensity was maximum in *B. vulgaris*. Coarse root biomass ranged from 0.6 kg m^−3^ in *B. nutans* to 2.0 kg m^−3^ in *B. vulgaris* and *B. bambos*. Fine root biomass ranged from 1.1 kg m^−3^ in *B. nutans* to 4.5 kg m^−3^ in *D. hamiltonii*. Contribution of fine roots in terms of intensity and biomass was much higher than coarse roots. Fine root biomass showed declining trend with increase in soil depth in all the species. During sixth year, the litter fall ranged from 8.1 Mg ha^−1^ in *D. stocksii* to 12.4 Mg ha^−1^ in *D. hamiltonii*. Among soil physical properties significant improvement were recorded in hydraulic conductivity, water stable aggregates and mean weight diameter. Soil pH, organic carbon and available phosphorus under different species did not reveal any significant changes, while significant reduction was observed in total nitrogen and potassium. Significant positive correlation was observed between WSA and iron content. Soil microbial population and enzyme activities were higher in control plot. Considering root distribution, biomass, soil hydraulic conductivity and water stable aggregates, *B. bambos*, *B. vulgaris* and *D. hamiltonii* are recommended for rehabilitation of degraded lands prone to soil erosion.

## Introduction

Bamboo grows worldwide in at least 37 million ha and covers 3.2 percent of forest areas of their host countries, or about 1 percent of the global forest area^1^. Bamboo being an annual yielding crop with multipurpose utility has proven potential for contributing to poverty reduction, environmental protection and achievement of United Nations Sustainable Development Goals – SDG1, SDG7, SDG 11, SDG 12, SDG 13, SDG 15, and SDG 17^2^. Many countries are now exploring options for achieving land degradation neutrality through the use of bamboo. Bamboo also contributes to the Bonn Challenge, the global initiative targeting the restoration of 200 million hectares of the planet’s degraded lands, to which International Bamboo and Rattan Organization (INBAR) member states have agreed to contribute about five million hectares of bamboo plantations^3^.

India is the second richest country of the world, after China, in terms of bamboo genetic resources^4^. The bamboo area of the country is estimated to be 15.69 million hectare with total standing stock of 189 million tons^5^. Raising of bamboo on degraded soils improves soil quality and sequesters carbon in the soil^6,7^. Bamboo due to its fast growth and extensive root system improves soil physical, chemical and biological properties; controls soil erosion; filters sediment and is considered suitable for rehabilitation of degraded lands within a short period of time^8–11^. Its habit of producing new culms annually from underground rhizomes allows sustainable annual harvesting without disturbing the soil^10^.

On degraded soils, litter fall and fine roots of bamboo adds considerable amount of carbon and nutrients to the soil, which helps in improving soil health^11–13^. Root density and biomass are key indicators for measuring root system efficiency^14^. Worldwide, majority of the studies on root distribution are on different tree species^15–20^ and monopodial bamboo in China^10,21,22^. In sympodial bamboo, there is limited information mainly due to methodological complexities^23–25^. The present study, therefore, aims at understanding the rooting pattern of different bamboo species and its implications on soil properties. The study is a part of a major research project that aims to identify potential bamboo species for soil and water conservation and designing appropriate resource conservation measures for optimizing productivity from degraded lands in Himalayan foothills. The study hypothesized that the rooting behaviour of bamboo and its impact on soil properties are species specific.

## Material and Methods

### Study site

**Figure 1:**
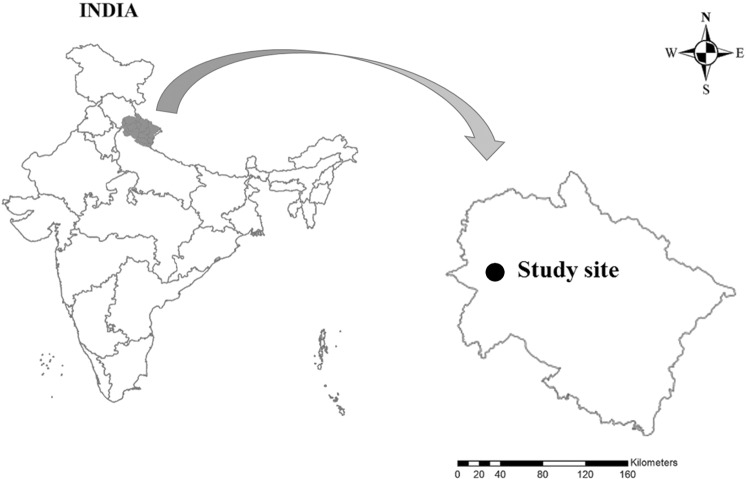
Location map of the study area.

The study was carried out at Dhulkot Research farm of ICAR-Indian Institute of Soil and Water Conservation, Dehradun, India located at 30°20′59′′ N latitude, 77°53′05′′ E longitude at 548 m above m.s.1 during the year 2012–2018 (Fig. 1). The region has agro-ecological conditions typical of the Doon valley and lower Himalayan ranges. The experiment soil belongs to Dhulkot series (Inceptisols) which are derived from heavy-textured, deep alluvium, yellowish-brown to dark yellowish-brown in colour, with few gravel and coarse rock fragments^26^.

The land use of the experiment was converted from forest to agricultural land about 65 years ago^26^. Due to deforestation and continuous intensive cultivation, the land has low to medium organic carbon (0.63–0.73% at study site vs 2.04% in adjoining forest), high bulk density (1.43–1.5 Mg m^−3^ at study site vs 1.20 Mg m^−3^ in adjoining forest), low hydraulic conductivity (0.9–1.8 cm hr^−1^ at study site vs 2.78 cm hr^−1^ in adjoining forest) and low pH (5.35–5.95 at study site vs 6.2 in adjoining forest)^27^. Mechanical analysis revealed that the soil contains 37% silt, 40% sand and 23% clay, and belongs to silty clay loam type. Long term average annual rainfall (last six decades) recorded is 1660 mm, out of which 82% is received during the monsoon months from June to September. Mean maximum temperature is recorded as 37 °C in the months of May and June, and mean minimum temperature is 4 °C in the months of December and January.

### Experimental setup

Seven bamboo species viz., *Bambusa balcooa, B. bambos, B. vulgaris, B. nutans, Dendrocalamus hamiltonii, D. stocksii* and *D. strictus* were planted at spacing of 5 m x 4 m in July 2012. The experiment was laid out in Randomized Complete Block Design (RCBD) with three replications. For each species, 9 plants were planted in each plot as block plantation, which covers an area of 180 m^2^. In total there are 21 plots covering an area of 3780 m^2^. All the selected species are of commercial importance and on priority list of National Bamboo Mission, Government of India and International Bamboo and Rattan Organization (INBAR). The fields selected for this study were ploughed twice using a disc harrow and then levelled. Pits of 0.45 cm^3^ size were dug manually. One year old nursery raised plants were transplanted in pits in July 2012. Any plants that died were replanted immediately. The experiment was conducted under rainfed conditions and no irrigation was done till 6^th^ year. The plantation raised for the experiment was maintained regularly for management of weeds by undertaking ploughing by tractor-drawn implements once every year. In addition, mounding operation (heaping of soil near base of clump) was done every year to provide support to the new culms which were produced annually. No manuring and fertilizer application was done till 6 years of age.

### Estimation of root intensity and biomass

Root sampling was done during first week of October 2017. Three uniform and healthy clumps were selected for each species for studying root distribution pattern. Depth wise root distribution was studied by profile wall method^24,28^. This method provides a relatively quick and detailed quantitative distribution of roots and involves digging of trenches (4 m length, 1 m width, and 70 cm depth) at 2 m distance on both sides of the central row of the clump. Keeping in view the large amount of excavation, the number of clumps for each species was restricted to three. Thus, in total 21 clumps (7 species × 3 replications) were selected for the excavation work. The walls of the trenches were made smooth by removing 1–2 cm soil from the soil surface to bottom of the trench. Roots which protruded from the working face of the profile wall were cut using sharp knife. After exposing the walls, an iron frame of 50 cm × 60 cm consisting of square grids of 10 cm× 10 cm size was placed against the profile wall. The number of roots was counted directly with in the frame on the wall at depths of 0–10, 10–20, 20–30, 30–40, 40–50 and 50–60 cm. The same procedure was repeated by placing the iron frame at 50 cm intervals along the length of trench on both sides. The counted roots were classified into coarse (>2.5 mm diameter) and fine (<2.5 mm diameter) roots. Root counts from 10 cm × 10 cm square grid were converted into rooting intensity i.e. number of roots m^−2^.

For root biomass estimation, soil cores were taken from the area around each clump at 4 different positions by driving a sharp edged core sampler into the soil to a depth of 0–10, 10–20, 20–30, 30–40 and 40–50 cm. Soil cores were placed in polythene bags and transported to the laboratory. All the samples were soaked in water for 8 hours and then manually stirred. The soil root suspension was passed through a sequence of sieves for categorising them into coarse roots (>2.5 mm diameter) and fine roots (<2.5 mm diameter). The process of washing was repeated several times before the soil slurry was discarded. All the roots were oven dried to a constant weight at temperature of 65 ± 2 °C. Litter production was monitored from the year 2015 to 2017 using litter traps of 1× 1 m size placed in the centre of the four clump in each replication.

### Soil properties

Composite soil samples were collected from surface soil (0–30 cm) using soil auger at the time of plantation for determining the changes of soil chemical properties. The collected samples were air-dried and ground to pass through a 2-mm sieve. Soil organic carbon (OC) content was determined by the Walkley and Black, Method^29^. Total nitrogen (TN) was determined by Kjeldahl Method^30^. Available P was determined colorimetrically by the Olsen method and extracted K by flame photometry. Soil pH was measured in soil suspension (1:2.5) using pH electrode^31^. Ethylenediaminetetraacetic acid (EDTA) titration method was used for measuring calcium and magnesium. Available micronutrients were extracted with DTPA extractant and determined using inductively coupled plasma (ICP) mass spectroscopy. Soil samples collected during the year 2018 under different bamboo species were analysed for soil physical, chemical and microbial properties. The values of soil pH, organic carbon, NPK in the year 2018 were compared with the initial values (2012) to get more reliable results. Soil physical, secondary nutrients, micronutrients, microbial and enzymatic activities were compared with samples collected from nearby control plots as baseline data of year 2012 was not available. Control plots were maintained in the same field away free from the effect of different bamboo species in undisturbed condition and served as reference for assessing the changes for soil physical, microbial and enzymatic activities. Soil bulk density was measured by the Core Method^32^. Saturated hydraulic conductivity (Ks) was measured by the Constant Head Method. The aggregate size distribution was determined by wet sieving method^33^.

Soil was also analysed for total microbial counts by Standard Pour Plate Technique. Soil microbial population isolation was done using Serial Dilution Method^34^ and dilution spread on Nutrient Agar, M001 (bacteria), Potato Dextrose Agar, M096 (fungi) and Actinomycetes isolation agar, M490 (actinomycetes) medium. All media was procured from Himedia, Mumbai India. The population was expressed as colony forming units per gram of soil (cfu/g soil). Phosphtase enzyme estimation was carried out by method given by Tabatabai and Bremner^35^. Dehydrogenase enzyme estimation was carried out by method given by Casida *et al*.^36^. β-glucosidase activity was determined using same method applied for acid and alkaline phosphatase activity, with the modification that the substrate was p-nitrophenyl-glucopyranoside^37^.

### Statistical analysis

Descriptive statistical analysis was carried out and range of variability and deviation in different parameters were established using standard error of mean. The Randomized Complete Block Design (RCBD) with three replications was used to compare treatment effects between species. For determining significant difference in treatment means, post-hoc test viz., Tukey’s Honest Significant Difference (HSD) was used at 0.05 level of significance. While analysing percentage data, Arc Sine transformation was carried to normalize the data before analysis. Soil chemical properties (pH, OC, NPK) were analysed using paired t-test to know whether bamboo plantation actually changed soil chemical properties from initial value. For comparing the values of micronutrients content of soils instead of paired t-test the mean and standard deviation values were considered. All the statistical analysis was carried out using SAS 9.3 software.

## Results

### Root distribution

**Figure 2:**
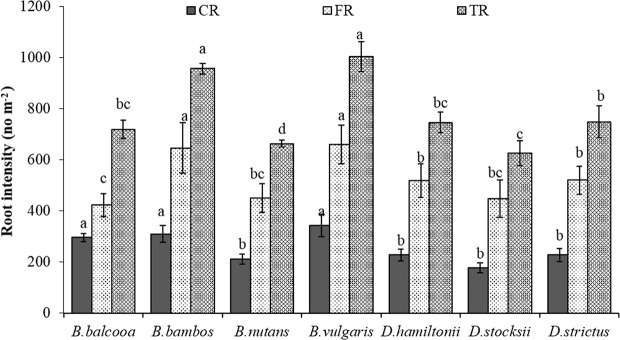
Root intensity (average of 0–60 cm) in different bamboo species. Error bars are standard deviations. Different letters indicate significant differences (P < 0.05) between species.

Average coarse root intensity (CRI) irrespective of soil depth showed significant variations in different species (F = 12.68, P < 0.001). Distribution of CRI for different species is given in Fig. 2. CRI was maximum in *B. vulgaris* (343.3 no. m^−2^) which was statistically at par with *B. bambos* (310.5 no m^−2^) and *B. balcooa* (296.6 no. m^−2^). Least CRI was observed in *D. stocksii* (177.78 no. m^−2^) which was at par with *D. hamiltonii* (227.7 no m^−2^) and *D. strictus* (227.7 no. m^−2^). Selected bamboo species had significant effect (F = 7.04, P < 0.001) on average fine root intensity (FRI) irrespective of soil depth (Fig. 2). Highest FRI was recorded in *B. vulgaris* (660 no. m^−2^) which was statistically similar to *B. bambos* (646.7no m^−2^). Minimum FRI was recorded in *B. balcooa* (423.3 no m^−2^) which was statistically at par with *D. stocksii* (448.3 no. m^−2^) and *B. balcooa* (423.3 no. m^−2^). Average total root intensity (TRI) irrespective of soil depth (Fig. 2) also varied significantly in different species (F = 10.83, P < 0.001) and maximum TRI was recorded in *B. vulgaris* (1003 no. m^−2^) which was statistically at par with *B. bambos* (957.3). The minimum TRI (626 no. m^−2^) was recorded in *D. stocksii*.

**Figure 3:**
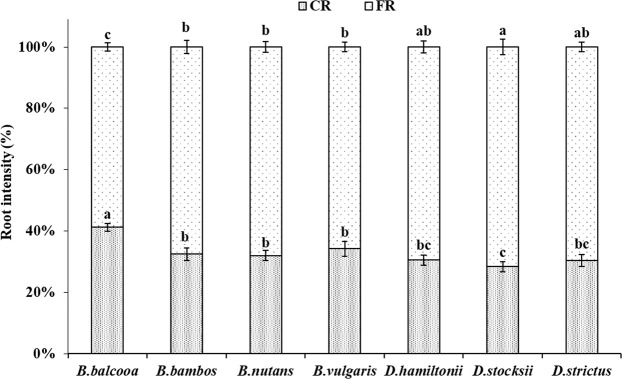
Distribution of coarse and fine root intensity in different bamboo species. Error bars are standard deviations. Different letters indicate significant differences (P < 0.05) between species.

In all the species, the contribution of fine roots (FRs) was more as compared to coarse roots (CRs) (Fig. 3). The FRs contribution was statistically lowest (58.7%) in *B. balcooa* and highest (71.6%) in *D. stocksii*. The percentage of FR to CR was statistically at par (69.5%) for *D. hamiltonii* and *D. strictus* (68%), *B. bambos* (67.6%)*, B. nutans* (67.9%) and *B. vulgaris* (65.7%) at 5% level of significance (Fig. 3).

**Figure 4:**
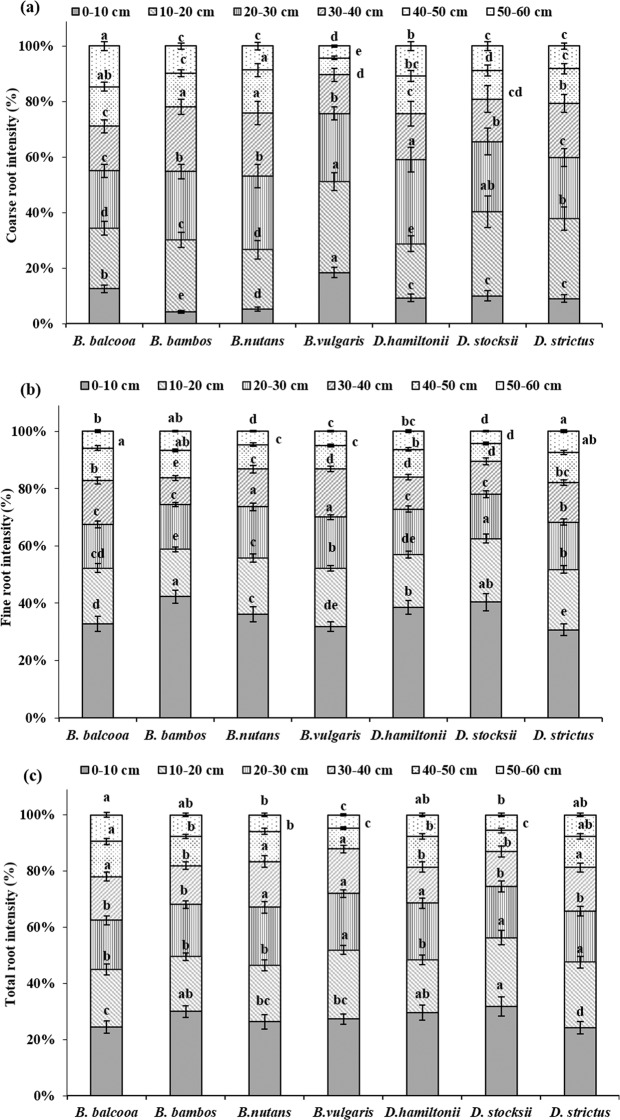
Depth wise distribution of root intensity in different bamboo species. Error bars are standard deviations. Different letters indicate significant differences (P < 0.05) between species.

Depth wise distribution of CRI in bamboo showed significant variation at all soil depths except at 40–50 cm, where no significant differences (P > 0.05) were observed (Table 1). Depth wise distribution of CRI in all the species revealed that surface horizon (0–10 cm) registered less CRI and the underlying 10–20 cm and 20–30 cm layer had more root counts (Table 1). Significant higher CRI was recorded in *B. vulgaris* at 0–10, 10–20 and 20–30 cm soil depths (Table 1). Depth wise distribution of CRI (Fig. 4a) further revealed that in *B. vulgaris*, maximum 76% CRs were distributed in 0–30 cm soil layer, which was followed by *D. stocksii* (65%). In rest of the species, the contribution of CRs in 0–30 cm soil depth varied from 53–60% (Fig. 4a).

**Table 1:** Root intensity (no. m^−2^) in different bamboo species at various soil depths.

Species	0–10 cm	10–20 cm	20–30 cm	30–40 cm	40–50 cm	50–60 cm
**Coarse root intensity**						
*B. balcooa*	223.3b	390.0bc	366.7abc	286.7b	253.3a	260.0a
*B. bambos*	80.0c	481.7b	460.0ab	435.0a	225.0a	181.7ab
*B. nutans*	66.7c	273.3c	340.0bc	290.0b	196.7a	110.0bc
*B. vulgaris*	380.0a	673.3a	506.7a	286.7b	126.7a	86.7c
*D. hamiltonii*	126.6bc	266.7c	413.3abc	226.7bc	186.7a	146.7bc
*D. stocksii*	106.7c	323.3bc	270.0c	163.3c	110.0a	93.3bc
*D. strictus*	123.3bc	393.3bc	300.0c	266.7b	173.3a	110.0bc
F value	9.72	7.27	3.33	9.11	NS	4.3
P value	0.001	0.002	0.036	0.001	NS	0.015
**Fine root intensity**						
*B. balcooa*	833.3c	493.3d	390.0c	386.7b	286.7a	150.0a
*B. bambos*	1640.0a	640.0bc	606.7ab	360.0b	373.3a	260.0a
*B. nutans*	980.0bc	526.7 cd	486.7bc	356.7b	230.0a	126.7a
*B. vulgaris*	1260.0b	806.7a	706.7a	666.7a	320.0a	200.0a
*D. hamiltonii*	1200.0b	573.3bcd	493.3bc	346.7b	300.0a	200.0a
*D. stocksii*	1086.7bc	596.7bcd	416.7c	306.7b	166.7a	116.7a
*D. strictus*	960.0bc	656.7b	516.7bc	430.0b	326.7a	233.3a
F value	6.89	6.36	3.33	3.60	NS	NS
P value	0.002	0.003	0.036	0.028	NS	NS
**Total root intensity**						
*B. balcooa*	1056.7c	883.3b	756.7c	673.3bc	540.0a	410.0a
*B. bambos*	1720.0a	1121.7b	1066.7ab	795.0ab	598.3a	441.7a
*B. nutans*	1046.7ab	800.0b	826.7c	646.7bc	426.7a	236.7a
*B. vulgaris*	1640.0c	1480.0a	1213.3a	953.3a	446.7a	286.7a
*D. hamiltonii*	1326.7bc	840.0b	906.7bc	573.3bc	486.7a	346.7a
*D. stocksii*	1193.3c	920.0b	686.7c	470.0c	276.7a	210.0a
*D. strictus*	1083.3c	1050.0b	816.7c	696.7abc	500.0a	343.3a
F value	5.84	4.73	5.95	3.30	NS	NS
P value	0.005	0.011	0.004	0.037	NS	NS

Within soil depth, different letters indicate significant differences (P < 0.05) between the species.

Depth wise distribution of FRI in different bamboo species revealed statistically significant differences up to 40 cm soil depth, after which (40–60 cm) no significant differences were recorded between different species at P = 0.05 (Table 1). FRI further depicted a declining trend with increase in soil depth for all the species. At 0–10 cm soil depth FR intensity (no. m^−2^) varied significantly (F = 6.89, P = 0.002) for different species (Table 1). Maximum and minimum FR intensity at 0–10 cm soil depth was recorded in *B. bambos* (1640 no. m^−2^) and *B. balcooa* (833.3 no. m^−2^), respectively. At 10–20 cm soil depth, significant highest FRI (F = 6.36 P = 0.003) was observed in *B. vulgaris* (806.7 no m^−2^), while values for *D. stocksii, B. nutans* and *D. hamiltonii* were at par. Figure 4b revealed that in all the species, majority of finer roots were distributed in 0–30 cm soil layer with maximum 78% in *D. stocksii* and minimum 68% in *B. balcooa*.

Depth wise distribution of total rooting intensity (TRI) showed significant variations in different bamboo species from 0–40 cm soil depth (Table 1). No significant differences were recorded in TRI from 40–60 cm soil depth at 5% level of significance. Except *B. nutans* and *D. hamiltonii*, TRI was highest in 0–10 cm soil depth for all species which declined with increase in soil depth. Figure 4c revealed that about 24–32% roots were distributed in 0–10 cm soil layer in different species, whereas in 0–30 cm soil layer, about 62–75% roots was distributed for different species.

**Figure 5:**
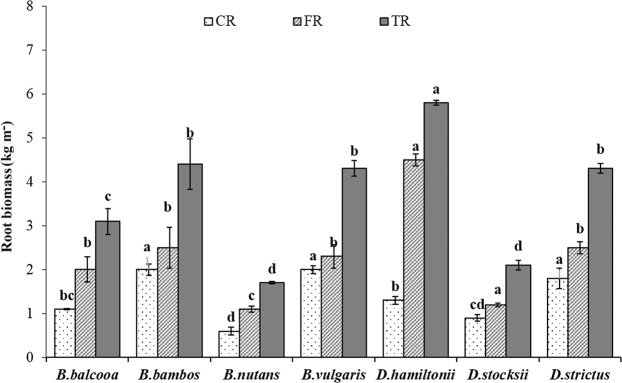
Root biomass (Kg m^−3^) in different bamboo species. Error bars are standard deviations. Different letters indicate significant differences (P < 0.05) between species.

**Figure 6:**
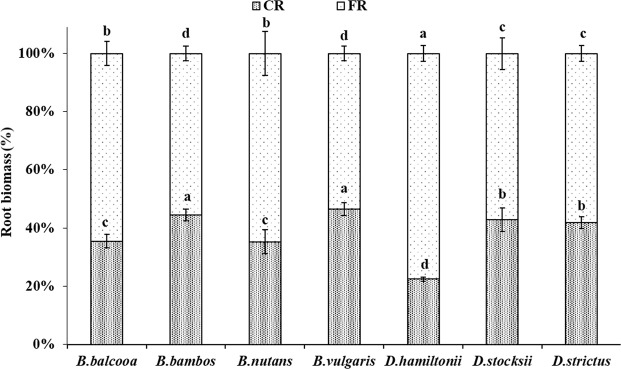
Root biomass (%) in different bamboo species. Error bars are standard deviations. Different letters indicate significant differences (P < 0.05) between species.

Coarse root biomass (total upto 50 cm soil depth) revealed significant statistical variation (F = 15.90, P < 0.001) among different bamboo species (Fig. 5). Coarse root biomass (CRB) ranged from 0.6 kg m^−3^ in *B.nutans* to 2.0 kg m^−3^ in *B. vulgaris* and *B. bambos,* which however, was statistical at par with *D. strictus* (1.8 kg m^−3^). Fine root biomass (total upto 50 cm soil depth) varied significantly in different species (F = 22.5, P < 0.001). Statistically highest (4.5 kg m^−3^) and lowest (1.1 kg m^−3^) fine root biomass (FRB) was recorded in *D. hamiltonii* and *B. nutans*, respectively. The values of FRB in *B. balcooa, B. bambos, B. vulgaris* and *D. strictus* were statistically alike. Like CRB and FRB, total root biomass (TRB) also varied significantly in different species (F = 27.40, P < 0.001). Significantly highest TRB (5.8 kg m^−3^) was observed in *D. hamiltonii,* whereas, lowest TRB was recorded in *B. nutans* (1.7 kg m^−3^) which was statistically at par with *D. stocksii* (2.1 kg m^−3^) at 5% level of significance. Compared to coarse root biomass, the contribution of fine roots biomass was higher in all the species (Fig. 6). The FR contribution was statistically highest (77%) in *D. hamiltonii* and lowest (53.3%) in *B. vulgaris,* which was at par with *B. bambos* (55.6%).

**Figure 7:**
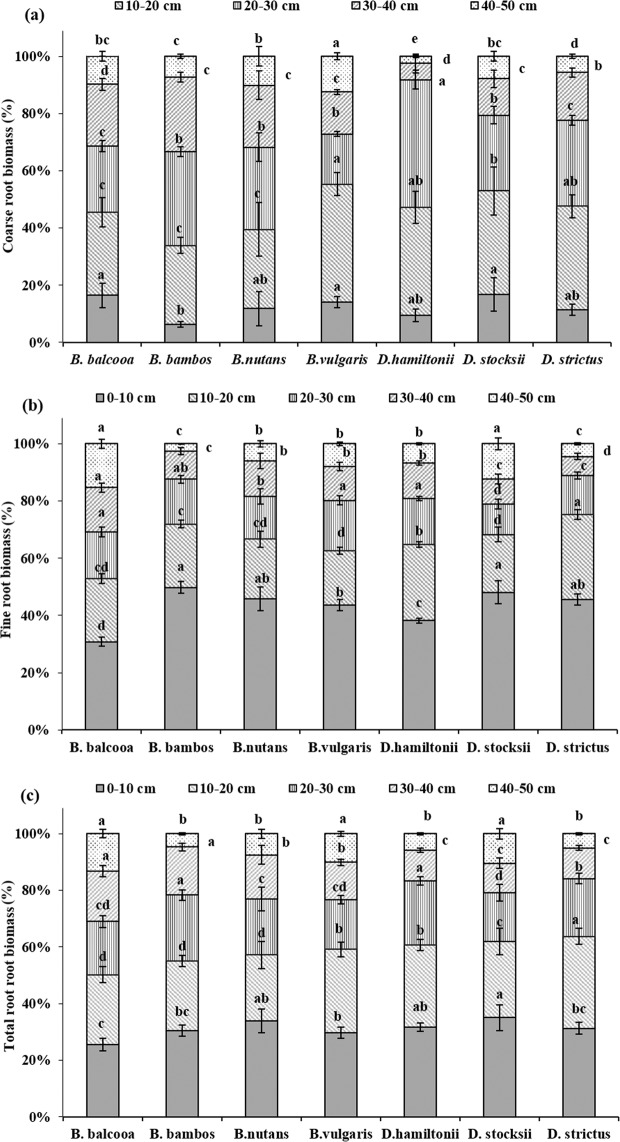
Depth wise distribution of root biomass in different bamboo species. Error bars are standard deviations. Different letters indicate significant differences (P < 0.05) between species.

Depth wise distribution of CRB revealed non-significant variations at 0–10 cm and 40–50 cm soil depths in different species (Table 2). At 10–20 cm soil depth, species had significant effect on CRB (F = 13.23, P < 0.001) and highest CRB was recorded in *B. vulgaris* (838.6 g m^−3^). At 20–30 cm soil depth, significant higher CRB was observed in *B. bambos* (645.4 g m^−3^), which was statistically at par with *D. hamiltonii* (600.2 g m^−3^). Maximum concentration of CRB was observed at 10–20 and 20–30 cm soil depths (Fig. 7a). Both the soil profiles collectively accounted for 52% biomass in *B. balcooa*, 56% in *B. nutans*, 59% in *B. vulgaris*, 60% in *B. bambos*, 63% in *D. stocksii*, 66% in *D. strictus,* and 82% in *D. hamiltonii*.

**Table 2:** Root biomass (g m^−3^) in different bamboo species at various soil depths.

Species	0–10 cm	10–20 cm	20–30 cm	30–40 cm	40–50 cm
**Coarse root biomass**					
*B. balcooa*	186.8a	331.2 cd	263.3bc	246.3bcd	110.4a
*B. bambos*	123.4a	540.8b	645.4a	509.6a	144.4a
*B. nutans*	69.2a	163.0d	168.9c	127.4c	59.4a
*B. vulgaris*	287.6a	838.6a	356.7bc	297.9b	254.8a
*D. hamiltonii*	127.4a	508.9bc	600.2a	76.4d	33.8a
*D. stocksii*	144.7a	314.2d	229.3c	111.0 cd	67.9a
*D. strictus*	203.8a	644.9b	535.0ab	297.2bc	101.9a
F value	NS	13.23	6.35	6.15	NS
P value	NS	< 0.001	0.003	0.004	NS
**Fine root biomass**					
*B. balcooa*	614.3 cd	442.0bcd	325.5b	308.6b	305.7a
*B. bambos*	1222.9b	543.5bc	385.0b	243.5b	62.3a
*B. nutans*	502.2d	227.2d	163.5b	135.9b	65.8a
*B. vulgaris*	996.4bc	435.9bcd	401.9ab	271.7b	181.2a
*D. hamiltonii*	1715.5a	1189.0a	721.9a	552.0a	305.7a
*D. stocksii*	588.8d	247.1 cd	130.2b	107.6b	150.0a
*D. strictus*	1126.7b	734.3b	336.9b	164.2b	110.4a
F value	11.20	11.31	3.26	4.34	NS
P value	<0.001	<0.001	0.039	0.015	NS
**Total root biomass**					
*B. balcooa*	801.1c	773.2 cd	588.8 cd	554.8ab	416.1a
*B. bambos*	1346.3b	1084.3bc	1030.4ab	753.0a	206.7a
*B. nutans*	571.4c	390.1e	332.4d	263.3bc	125.3a
*B. vulgaris*	1284.1b	1274.6b	758.7bcd	569.6a	436.0a
*D. hamiltonii*	1842.9a	1697.9a	1322.1a	628.5a	339.6a
*D. stocksii*	733.5c	561.4de	359.5d	218.6c	217.9a
*D. strictus*	1330.5b	1379.2a	871.9bc	461.4abc	212.3a
F value	10.62	18.41	6.40	3.80	NS
P value	<0.001	<0.001	0.003	0.023	NS

Within soil depth, different letters indicate significant differences (P < 0.05) between the species.

Significant species variations were observed in FRB at different soil depths except 40–50 cm (Table 2). *D. hamiltonii* depicted statistically higher FRB at all the soil depths. FRB showed declining trend with increase in soil depth in all the species with surface horizon (0–10 cm) depicting highest values. *B. nutans* registered significant lower FRB in all the soil depths except 20–40 cm. Distribution of FRB revealed that maximum concentration of biomass was observed at 0–10 cm soil depth for all the species (Fig. 7b). It accounted for minimum 31% biomass in *B. balcooa* to highest 50% in *B. bambos* which were significantly different at 5% level of significance. Results further revealed that top 0–30 cm soil accounted for 69% biomass in *B. balcooa*, 79% in *B. vulgaris* and *D. stocksii*, 81% in *D. hamiltonii* and *B. nutans*, 88% in *B. bambos* and 89% in *D. strictus*.

Significant species variations were observed in TRB at different soil depths, except 40–50 cm (Table 2). Effect of species was significant on TRB at 0–10 cm (F = 10.62, P < 0.001), 10–20 cm (F = 18.41, P < 0.001), 20–30 cm (F = 6.40, P = 0.003) and 30–40 cm (F = 3.80, P = 0.023) soil depth. *D. hamiltonii* depicted significant higher TRB at 0–30 cm soil depth, whereas at 30–40 cm, highest TRB was recorded in *B. bambos*. TRB was highest in 0–10 cm soil depth for all the species. *B. nutans* registered significantly lowest total root biomass in all the soil depths, except 30–40 cm. Distribution pattern revealed that maximum FRB was at 0–10 cm soil depth for all the species (Fig. 7c). *D. stocksii* accounted for maximum 35% biomass in this soil layer while lowest of 26% was observed in *B. balcooa* and they were statistically different at 5% level of significance. Results further revealed that top 0–30 cm soil accounted for maximum 84% in *D. strictus*, which was statistically at par with *D. hamiltonii*.

### Litter production

**Figure 8:**
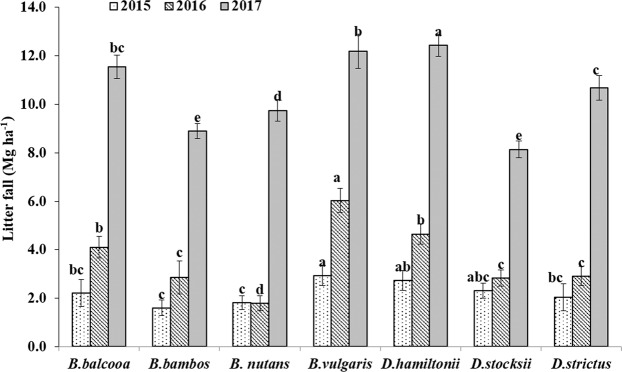
Litter fall (Mg ha^−1^) in different bamboo species. Error bars are standard deviations. Different letters indicate significant differences (P < 0.05) between species.

Litter production depicted increasing trend with increase in age of plantation (Fig. 8). Analysis of Variance (ANOVA) revealed that, effect of species on litter fall was significant in all the years with F value 4.26 (P = 0.016), 32.1 (P < 0.001) and 32.80 (P < 0.001) for 2015, 2016 and 2017, respectively. In the years 2015 and 2016, significantly higher litter fall was recorded in *B. vulgaris* with values of 2.94 and 6.04 Mg ha^−1^ respectively, while lowest litter fall was observed in *B. nutans* with values of 1.81 and 1.79 Mg ha^−1^, respectively. In the year 2017, litter fall increased significantly and reached to maximum value of 12.4 Mg ha^−1^in *D. hamiltonii* which was followed by *B. vulgaris* (12.1Mg ha^−1^), *B. balcooa* (11.5 Mg ha^−1^), *D. strictus* (10.7 Mg ha^−1^) and *B. nutans* (9.7 Mg ha^−1^). Lowest litter fall in the year 2017 was recorded in *D. stocksii* (8.1 Mg ha^−1^), which was statistically at par with *B. bambos* (8.9 Mg ha^−1^).

### Soil properties

#### Physical properties

**Figure 9:**
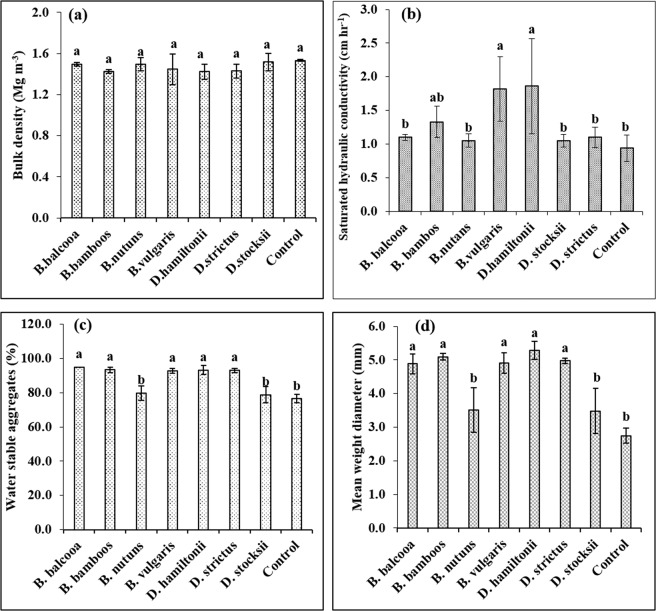
Changes in soil physical properties under different bamboo species. Error bars are standard deviations. Different letters indicate significant differences (P < 0.05) between species.

Bulk density (BD) did not show any significant variation under different species at P = 0.05; though slight reduction was recorded as compared to control under different bamboo species. Least BD of 1.42 Mg m^−3^ was observed under *D. hamiltonii* and *B. bambos* (Fig. 9a). Saturated hydraulic conductivity (Ks) showed significant variation (F = 3.74, P = 0.017) under different bamboo species (Fig. 9b). The highest Ks was recorded under *D. hamiltonii* (1.86 cm hr^−1^) which however statistically at par with *B. vulgaris* (1.82 cm hr^−1^). Lowest Ks (0.95 cm hr^−1^) was observed under control plot (reference site) which, however, was statistically at par with *D. strictus* (1.1 cm hr^−1^)*, B. nutans* (1.05 cm hr^−1^), *D. stocksii* (1. 050 cm hr^−1^) and *B. balcooa* (1.10 cm hr^−1^).

Water stable aggregates (WSA) varied significantly (F = 7.26, P = 0.001) under different bamboo species (Fig. 9c,d). The values of WSA were highest in *B. balcooa* (94.9%), which however was statistically at par with *B. bambos* (93.4%)*, D. hamiltonii* (93.2%), *D. strictus* (93.0%) and *B. vulgaris* (92.8%). Lowest WSA (76.4%) were recorded in control plot (reference site) which, however, was statistically at par with *D. stocksii* (78.8%) and *B. nutans* (79.7%). Mean weight diameter also showed significant variations (F = 6.09, P = 0.002) under different species. Significant higher MWD was recorded in *D. hamiltonii* (5.28), which was followed by *B. bambos* (5.09 mm), *D. strictus* (4.98 mm), *B. vulgaris* (4.91 mm) and *B. balcooa* (4.89 mm). Values of MWD in *B. nutans* and *D. stocksii* were statistically at par with control plot.

#### Chemical properties

As compared to initial values, soil pH reduced slightly under all the species except *B. bambos* and *D. stocksii* (Table 3), though the changes were statistically non-significant at 95% confidence interval. The soil pH under control plot (6.25) was higher than all other bamboo species. Soil organic carbon (SOC) was reduced in all the species as compared to initial value, but only in case of *D. strictus* the change (0.73% to 0.53%) was significant with paired t value 4.33 (P = 0.05). Change (reduction) of total nitrogen as compared to initial value was significant in *B. bambos* (t = 5.49, P = 0.03), *B. nutans* (t = 7.12, P = 0.02), *D. hamiltonii* (t = 11.30, P = 0.01) and *D. strictus* (t = 9.61, P = 0.01). In all other species the decrease was non-significant at 5% level of significance. Soil phosphorus increased in *B. balcooa, B. bambos, B. vulgaris* and *D. hamiltonii* and decreased in *D. strictus, D. stocksii* and *B. nutans*. However, the changes were non-significant at 5% confidence interval. (Table 3). Potassium content was significantly reduced under all the species, except *B. nutans* where the decrease was significant at P < 0.05 (Table 3).

**Table 3:** Changes in pH, organic carbon and primary nutrients under different bamboo species.

Species	Statistics	pH		OC (%)		N (%)		P (ppm)		K (ppm)	
		Initial	2018	Initial	2018	Initial	2018	Initial	2018	Initial	2018
*B. balcooa*	Average	5.70	5.83	0.687	0.576	0.085	0.08	5.55	7.60	85.30	49.80
	Paired T Value	0.56(0.63)		−1.91(0.2)		−3.59(0.07)		2.42(0.14)		−11.31(0.01)	
*B. bambos*	Average	5.65	5.54	0.639	0.578	0.076	0.069	7.41	7.78	85.20	55.20
	Paired T Value	−1.36(0.31)		−0.69(0.56)		−5.49(0.03)		0.39(0.73)		−4.84(0.04)	
*B. nutans*	Average	5.85	5.48	0.647	0.565	0.063	0.054	8.06	7.60	83.2	63.10
	Paired T Value	−2.00(0.18)		−1.88(0.2)		−7.12(0.02)		−0.75(0.53)		−2.23(0.16)	
*B. vulgaris*	Average	5.65	5.38	0.688	0.682	0.073	0.074	6.07	6.82	84.50	50.70
	Paired T Value	−1.95(0.19)		−0.09(0.94)		0.58(0.62)		1.96(0.19)		−4.30 (0.05)	
*D. hamiltonii*	Average	5.95	5.66	0.715	0.591	0.079	0.065	5.65	6.06	81.70	53.40
	Paired T Value	−2.72(0.11)		−1.59(0.25)		−11.3(0.01)		0.42(0.71)		−7.21(0.02)	
*D. stocksii*	Average	5.35	5.81	0.632	0.612	0.079	0.078	6.97	6.69	92.00	52.20
	Paired T Value	2.31(0.15)		−0.43(0.71)		−0.42(0.71)		−0.56(0.63)		−4.74(0.04)	
*D. strictus*	Average	5.65	5.56	0.731	0.529	0.081	0.059	11.51	8.25	97.10	55.00
	Paired T Value	−0.42(0.72)		−4.33(0.05)		−9.61(0.01)		−1.55(0.26)		−5.67(0.03)	

Values in the parenthesis are P value of paired t test.

Significant variations were observed for secondary and micronutrients under different bamboo species (Table 4). Calcium was lowest under *B. nutans,* which however, was at par with *D. hamiltonii* and *D. strictus*. Highest calcium was recorded under *D. stocksii*. Magnesium content was also significantly reduced under *B. nutans*. The highest magnesium content was recorded under *B. vulgaris,* which was at par with *D. strictus* and *D. stocksii*. Iron content was highest under *B. bambos* while lowest iron content was recorded under control, which was at par with *B. nutans* and *D. stocksii* (Table 3). Zinc content was highest under *B. balcooa,* which was at par with *B. vulgaris*. Lowest zinc content was recorded under control. Zinc content in rest of the species was at par. Nickel content was highest in control plot, which was at par with *B. nutans*. Lowest nickel content was recorded under *B. vulgaris*.

**Table 4:** Secondary and micronutrients (ppm) under different bamboo species.

Species	Ca	Mg	Fe	Zn	Ni
*B. balcooa*	116.7bc	46.4c	39.86ab	4.67a	0.153b
*B. bambos*	145.3b	49.6c	42.60a	3.60b	0.120bcd
*B. nutans*	78.0c	31.6d	32.20c	3.73b	0.207a
*B. vulgaris*	109.3bc	94.0a	35.27bc	4.80a	0.087d
*D. hamiltonii*	102.7c	27.3d	36.26bc	3.87b	0.093 cd
*D. strictus*	112.7c	91.9a	40.13ab	3.53b	0.127bcd
*D. stocksii*	188.0a	90.0a	33.67c	3.53b	0.140bc
Control	146.7b	66.1b	30.93c	2.60c	0.227a
F value	6.43	40.56	4.39	7.27	9.80
P value	0.02	<0.001	0.009	<0.001	<0.001

Different letters indicate significant differences between the species.

#### Soil microbial and enzymatic activities

**Figure 10:**
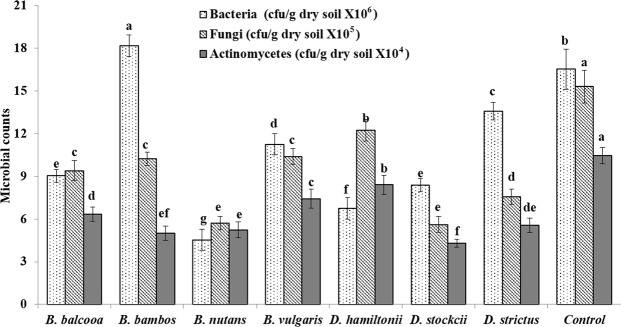
Changes in soil microbial properties under different bamboo species. Error bars are standard deviations. Different letters indicate significant differences (P < 0.05) between species.

Bacterial population (Fig. 10) showed significant differences under different species (F = 137.8, P < 0.001). Significantly highest bacterial count was observed in *B. bambos* followed by control (reference site). Least bacterial count was recorded in *B. nutans*. Species also had significant effect on fungal count (F = 82.6, P < 0.001) and highest fungal count was recorded in control (Fig. 10). Among different bamboo species, highest fungal counts were recorded in *D. hamiltonii* (12.2) and least in *D. stocksii* (5.7) which was at par with *B. nutans* (5.6). Fungal count in *B. balcooa, B. bambos* and B*. vulgaris* was statistically at par. Actinomycetes count (Fig. 10) varied significantly in different species (F = 58.9, P < 0.001). Significantly highest actinomycetes count was recorded in control (10.5) followed by *D. hamiltonii* (8.4) and *B. vulgaris* (7.4). Least actinomycetes count was recorded in *D. stocksii* (4.3).

Significant variations were observed in all the enzymatic activities under different bamboo species (Table 5). Dehydrogenase activity (DH) showed significant variations (F = 706, P < 0.001) and was highest in control plot (115.4). It reduced drastically under all the bamboo species with minimum in *D. stocksii* (10.9) which was at par with *B. bambos* (Table 5). β-glucosidase activity (BG) varied significantly in different species (F = 140.6, P < 0.001) and was significantly higher in control plot (Table 5). Among different bamboo species, significantly higher BG activity was recorded in *B. vulgaris* (149.8), which however, was at par with *B. balcooa* (144.7). Lowest BG activity was recorded under *D. stocksii* (108.7). Higher acid phosphatase activity (APA) was also observed in control (Table 5). Values of APA in *D. hamiltonii, D. strictus* and *B. balcooa* were statistically alike and the least APA activity was observed in *B. nutans* (164.1). Alkaline phosphatase activity (AlPA) was significantly (F = 300.2, P < 0.001) higher in control (Table 5) which was followed by *B. vulgaris* (67.2). Least alkaline phosphatase activity was found in *D. stocksii* (41.4).

**Table 5:** Soil enzymatic activities under different bamboo species.

Species	Dehydrogenase (µg TPF released/g of dry soil/24 hrs)	β-Glucosidase (µg PNP released/g of dry soil/hr)	Acidic phosphatase (µg PNP/g of dry soil/hr)	Alkaline phosphatase (µg PNP/g of dry soil/hr)
*B. balcooa*	47.0c	144.7b	301.3b	62.2c
*B. bambos*	14.3e	115.4ef	274.4c	45.4e
*B. nutans*	26.5d	122.5 cd	164.1e	53.0d
*B. vulgaris*	81.7b	149.8b	273.9c	67.2b
*D. hamiltonii*	22.3d	116.3de	300.5b	54.6d
*D. stockcii*	10.9e	108.7 f	248.0d	41.4 f
*D. strictus*	25.9d	128.0c	314.0b	62.8c
Control	115.4a	189.8a	348.6a	74.4a
F value	706.1	140.6	95.9	300.2
P value	<0.001	<0.001	<0.001	<0.001

Different letters indicate significant differences between the species.

## Discussion

### Root distribution

Root intensity and biomass showed significant variations in different species (Fig. 2). The difference in root intensity and biomass in different species may be attributed to biotic and abiotic factors, such as species composition, microenvironment, soil micro-organism, root herbivores and soil resource availability^38,39^. Numerous studies have indicated that cumulative belowground biomass and root life spans are controlled by root respiration, which is dependent on canopy assimilation^17,18,40,41^. Variation in root biomass, therefore, can be ascribed to variation in above ground growth characteristics of different bamboo species.

CRI and CRB were highest in *B. vulgaris* and *B. bambos*. Coarse roots are mainly for providing support to the culms. Higher coarse root intensity in *B. vulgaris, B. bambos* and *B. balcooa* indicates their usefulness in supporting the plant and providing slope stability more efficiently. Further, *B. bambos* due to thorny nature is not preferred by wild animals for grazing and browsing, and involves less care for its establishment in plantation programme, and thus can be suitably planted in the areas prone to biotic disturbances and soil erosion. Surface horizon (0–10 cm) had less CRI and CRB as compared to underlying 10–20 cm and 20–30 cm layer in all the species. Difference in rooting intensity in different soil layer may be attributed to variation in microenvironment at different soil layers. More roots at sub-surface depth may enable clumps to capture nutrients that would otherwise be leached from the upper horizons of the soil profile, which are in agreement with the findings of Divakara *et al*.^24^ and Kumar and Divakara^23^ who also reported highest root counts in the 10–20 cm layer with nearly 30% of total roots in 15 year old *B. bambos*. Results also revealed that majority of coarse roots were in 0–30 cm soil layer which is in agreement with the findings of Verma *et al*.^19^ who reported 68–87% coarse root within top 0–30 cm soil depth in different tree species. CRI and CRB were evenly distributed at different soil depths in *B. balcooa,* which indicates its suitability to support the soil at greater depth, particularly in light textured soil.

Average FRI and FRB also showed significant variation among different species with maximum value of FRI in *B. vulgaris,* which was statistically similar to *B. bambos*. FRB was higher (4.5 kg m^−3^) in *D. hamiltonii*. FRB was statistically at par in *B. bambos*, *B. vulgaris, B.balcooa* and *D. strictus*. FR contribute towards soil binding, absorption of nutrients and water^42–44^. Fine roots are considered exploitive and opportunistic in growth behavior, as they can rapidly adapt to changes in climate, nutrient and water supply^45^. Therefore, distribution, architecture, growth and morphology of fine roots are strongly influenced by micro environment^16,46^. The present values of FRB are within the reported range of 486–875 g m^−2^ yr^−1^ for bamboo in dry tropical Vindhyan region in India^47^.

Majority of FRI and FRB in all the species were distributed in 0–30 cm soil layer, which are in accordance with findings of Christanty *et al*.^13^ who reported that root systems of bamboo do not usually elongate to higher soil depth, but rather develop profuse mat of highly efficient fine roots within the uppermost soil layer. Fine roots due to rapid turnover provide carbon and nutrients, and improve soil health^15,48^. Higher fine root biomass is indicator of higher soil binding capacity. Exudation of organic substance formed from dead roots could bind micro aggregates and other particles into macro aggregates, which can improve soil anti-erosion capability^49^. Higher FRI in *B. vulgaris* and *B. bambos,* therefore, indicates better efficiency of species in binding soil particles, which may help in preventing soil erosion.

Vertical distribution of fine roots (FRI and FRB) showed declining trend with increase in soil depth in all the species with surface horizon (0–10 cm) depicting highest values. Vertical distribution of fine roots is mainly governed by myriad of endogenous (i.e., species genetic features) and exogenous factors, among which soil temperature, moisture, and nutrient availability are most dominant^42,50,51^. More FRs in upper soil layer in the present study can be attributed to easy availability of nutrients and water in the upper soil layer^48^. Large network of fine roots in upper layer not only implies the potential of specie to absorb soil nutrients, but also is helpful for onsite conservation of nutrients, particularly leachable elements like potassium that are intercepted and re-absorbed in the plant biomass. Zhou *et al*.^10^ and White and Childers^52^ also reported that 83% of the bamboo roots are present in top 0–30 cm of soil profile where they serve best in checking soil erosion. At 0–10 cm soil depth, maximum FRI and FRB was recorded in *B. bambos* and *D. hamiltonii* thus indicating that species might have higher capacity among tested species for uptaking soil moisture and nutrients from the soil. This may also enhance infiltration capacity of the soils that can be beneficial for *in-situ* rainwater conservation.

Litter production depicted increasing trend with increase in age of plantation. During sixth year, the litter fall ranged from 8.1 Mg ha^−1^ in *D. stocksii* to 12.4 Mg ha^−1^ in *D. hamiltonii*. The observed values of the litter fall were within the reported range by Tripathi and Singh^12^; Shamnughavel *et al*.^53^ and Kumar *et al*.^54^. Leaf litter protect the soil from the erosive impact of rain and improves soil health^9,12,13,53,55^ and thus *D. hamiltonii* can be considered good species for preventing soil erosion, improving soil health and checking evaporation losses from soil through mulch effect.

### Soil properties

Bulk density (BD) did not show any significant variations under different species; though slight reduction was recorded as compared to control under different bamboo species. Bulk density reflects the soil’s ability to function for structural support, water and solute movement, and soil aeration. High bulk density indicates soil compaction and low soil porosity which may cause restricted root growth, and poor air and water movement through the soil. Slight reduction in BD under bamboo as compared to control (without bamboo-reference site) may be attributed to higher fine roots production, turnover, litterfall, fauna, and other related biological processes^56,57^. Decrease in BD and compaction under trees was also reported by Mandal *et al*.^27,58^.

Saturated hydraulic conductivity (Ks) was higher under bamboo species as compared to control. The higher Ks values were observed under *B. vulgaris* and *D. hamiltonii*. Hydraulic conductivity of soil is basically the capacity of soil to let water pass through the pores or voids in the soil. High values of Ks indicate more permeability and low values indicate less permeability. Plant roots contribute to development of macropores by pushing through the soil while they grow or by leaving channels when they die. High Ks under *B. vulgaris* and *D. hamiltonii* may therefore be attributed to the higher fine root production (Tables 1 and 2; Figs. 2–7) and litter fall in these species, which may have resulted in a greater proportion of larger pores and better faunal activities that enhance Ks, preferential flow, and macropore flow^58,59,60^.

Water stable aggregates (WSA) and mean weight diameter (MWD) increased under all the studied bamboo species. Except *B. nutans* and *D. stocksii,* all the species were at par. Aggregate stability refers to the ability of soil aggregates to resist disintegration when disruptive forces associated with tillage and water or wind erosion are applied. WSA are critical for pore space, infiltration, microbial habitat, root growth, physical protection of organic matter and are the best index for evaluation of soil anti-erosion capability^61,62^. The mechanism behind aggregates includes fungal hyphae, bacteria and other soil organisms that bind soil particles by organic glues, which results from organic matter decomposition. Higher WSA in majority of bamboo species may, therefore, be attributed to protective covering of litter fall and dense fibrous root systems of bamboo species that acted as binding agents between particles, and hence resulted in larger-sized aggregates. Ekwue^63^ also observed a direct relation between the MWD of soils and litter fall. Saha *et al*.^64^ and Kukal *et al*.^65^ also reported higher MWD and macro-aggregates of soil under *Gmelina arborea, Parkia roxburghii, Michelia oblonga, Pinus kesiya* and *Populus deltoides* as compared to control plots. In addition, relationship between aggregate stability and soil chemical composition, particularly Ca and Mg contents of the soils, was also studied which indicated no direct relationship (Table 3). Therefore, it may be inferred that the fibrous root systems and litter fall is mainly responsible for making more stable aggregates as stated above. Sanchez^66^ also reported that reforestation with pines provided organic cements and fungal hyphae that reinforces soil aggregation and humus. 

As compared to initial values, soil pH reduced slightly under all the species, except *B. bambos* and *D. stocksii*, though the changes were non-significant. As compared to open (control), soil pH was reduced under all the species. Reduction in soil pH may be attributed to high leaf litter production (Fig. 8), whose decomposition might have produced organic acids, which in turn caused slight reduction in pH. Upadhyaya *et al*.^67^ studied two bamboo species and reported that soil under *Bambusa balcooa* was more acidic as compared to *Bambus palida* in north eastern part of India. Increase in soil pH under *B. bambos* and *D. stocksii* may be attributed to lower litter production by these species. Soil acidification due to continuous decomposition of organic matter under natural woodlands have also been reported^58,66^.

As compared to initial values, SOC was reduced under all the species except *D. strictus* though the changes were non-significant. The build-up of soil organic matter and nutrient turnover is affected by the input of nutrient through leaf, stem, branch and root. Differences in organic carbon content under various species may be due to addition of varying amounts of leaf litter, fine roots (Table 2; Figs. 2–7) and different decomposition rate. Our results are however, contrary with previous findings of various workers who reported improvement in soil quality and carbon sequestration under different bamboo species^6,7,11–13,68–69^. Reduction in SOC under all the species as compared to initial values under the present study may be attributed to initially lower leaf litter addition during establishment phase of the bamboo clumps (1–4^th^ year) due to which they could not exert a remarkable effect on SOC. Further, during establishment phase, the interspaces were ploughed mechanically, and mounding operation (heaping of soil near base of clump) was done annually to provide support to the new culms, which might also have resulted in loss of accumulated carbon to the atmosphere. Additionally, previous reports of soil improvement^6,7,11–13,68,69^ are from long term studies in 15–23 year old plantations where litterfall was high. In the present study, leaf litter production in all the species was less during initial phase but increased during 5^th^ year. It is assumed that the litterfall rate may increase further with increase in age and will result in carbon build up in the soil in the coming years. However, a long term monitoring of different soil properties is required to validate the above findings.

Significant reduction was observed for total nitrogen as compared to initial value in *B. bambos*, *B. nutans*, *D. hamiltonii* and *D. strictus*. Absorption of nutrients from the soil by bamboo depends on the growth habit and functionality of their roots and rhizomes^23^. Reduction under these species may be attributed to faster growth, higher uptake and storage of nutrients in the biomass and less turnover to the soil^55^. Singh and Singh^68^ also observed that the substantial amount of C and N were immobilized in microbial biomass due to litter fall in bamboo plantation, and the magnitude of immobilization increased with the age of plantation.

Soil phosphorus increased in *B. balcooa, B. bambos, B. vulgaris* and *D. hamiltonii* and decreased in *D. strictus, D. stocksii and B. nutans*, though the changes were non-significant. Increase in soil P may be attributed to higher rooting intensity and biomass in *B. bambos, B. vulgaris* and *D. hamiltonii* (Figs. 2 and 5), which might have caused dissolution of inorganic P of soils^70^. Mandal *et al*.^27,58^ reported that under natural wood stands, the extractable P is more than the corresponding control plots due to better rhizosphere environment created by the roots, which helps in dissolution of inorganic P. Reduction in phosphorus in *D. strictus, D. stocksii and B. nutans* may be attributed to to their high uptake of P by above ground biomass and less turnover through litter fall, which sometimes is responsible for immobilization of P in microbial biomass^68^. The non-significant variation in N and P in soil under different species may also be attributed to difference in accumulated biomass and leaf litter production in different species.

Available potassium in soil was significantly reduced under all the species, except *B. nutans*. Bamboo plays an important role in accumulation of K due to its ability of rapid uptake in biomass. The differential effect of bamboo species on soil available K may be due to the faster growth, higher K uptake and storage in biomass, and less turnover to the soil. Singh and Singh^68^ also observed that increasing demand by growing bamboo plants over the years also do not permit K to accumulate in topsoil. Rao and Ramakrishna^69^, Kumar *et al*.^54^ and Singh and Rai^71^ also reported relatively higher uptake of K by different bamboo species.

No trend was observed for calcium and magnesium under different bamboo species. As compared to control, iron content was significantly higher under all the bamboo species. *D. strictus* and *B. balcooa* depicted higher values for iron. Significant positive correlation was found between WSA and iron content (r = 0.839), and MWD and iron content (r = 0.814), which are in agreement with the findings of Barral *et al*.^72^ who reported direct relationship between structural stability and iron content. Higher WSA in *D. hamiltonii,* therefore, can also be attributed to combined effect of iron and organic carbon under this species. For zinc, higher values were observed under different bamboo species as compared to control. The higher values of Fe and Zn under bamboo may be attributed to addition through leaf litter and enrichment through fine root biomass. Nickel content, however, showed marked reduction under bamboo, which may be attributed to more of its uptake by the bamboo plants.

Microbial population revealed significant variations under difference species. Significantly highest bacterial count was observed in *B. bambos* followed by control (reference site). Fungal and actinomycetes counts were significantly highest in control. Soil microbial population plays key role in regulation of decomposition of soil organic matter, nutrient cycling and energy flow, and is indicator of soil maturity. Soil fungi aid in decomposition of organic matter in soils and are important in the processes of humus formation. Higher microbial count in control (without bamboo) may be attributed to no disturbance in these plots. Doran *et al*.^73^ and Allison *et al*.^74^ also reported higher microbial counts in no-till system which was not disturbed. Among different bamboo species, highest fungal counts were recorded in *D. hamiltonii* and least in *D. stocksii,* which was statistically at par with *B. nutans*. Significantly highest actinomycetes count was recorded in control followed by *D. hamiltonii* and *B. vulgaris.* Variation of microbial count under different bamboo species may be attributed to a variation in root intensity, biomass, quality and quantity of litter fall, FR turnover, and microclimatic environment of community^57,75^.

Enzymatic activities viz., dehydrogenase (DH), β-glucosidase (BG), acidic phosphatase (AP) and alkaline phosphatase (ALP) also revealed significant variations in different species. Control plot showed maximum enzymatic activities. Among different bamboo species, maximum DH, BG and AlP activities were observed in *B. vulgaris*, while AP activity was highest in *B. balcooa* and *D. hamiltonii*. Soil enzymes play an important role in organic matter decomposition and nutrient cycling and help to support biochemical processes which are essential for the maintenance of soil quality^76–78^. High DH and BG activity in control plot may be attributed to undisturbed soil, which might have created favourable environment for microorganisms as compared to bamboo where regular ploughing done. Dick *et al*.^76^ and Bergstrom *et al*.^79^ also reported higher enzymatic activity under undisturbed soil. DH reflecting oxidative activity of soil microflora is a good indicator of microbiological activity and is strongly correlated with SOC^80^. BG provides energy for microorganism and promotes organic matter decomposition. Higher DH and BG activities under *B. vulgaris* may, therefore, be attributed to high organic carbon content under the species as compared to other. Phosphatases are actively involved in phosphorus cycling by hydrolyzing organic P compounds to inorganic P. Higher phosphatase activity in *D. hamiltonii, B. balcooa* and *B. vulgaris* suggests that available P in these soils might be increased which is also evident from the results (Table 5). Overall, the variation in enzymatic activities under different bamboo species may also be attributed to obvious differences in microclimate, litter quality, quantity and root exudates^50,80,81^.

## Conclusion

The study revealed that root intensity and root biomass in different bamboo species varied considerably, which indicates that substantial benefits pertaining to resource conservation aspects in term of improved soil hydraulic conductivity, soil binding and aggregate stability can be achieved by selecting the appropriate bamboo species. *B. vulgaris, B. bambos* and *D. hamiltonii* due to higher fine root intensity, below ground biomass production and higher soil hydraulic conductivity can bind the soil more efficiently, and contribute to more ground water recharge. These species also performed better for improving physical properties of soil, and thus can be suitably grown for rehabilitation of degraded lands prone to soil erosion. *B. bambos* species due to thorny nature is also recommended for the areas prone to biotic disturbances and soil erosion. Not much significant improvement was observed on soil chemical and microbiological properties, which is attributed to low litter inputs of bamboo during initial stages to the soil. However, it is expected that with increase in age, higher litterfall may lead to a more carbon build up and improvement in soil fertility, for which monitoring needs to be done on long term. Research efforts are also required to study root mechanical properties, decay pattern of roots and biomass allometrics, which are important in understanding the mechanism of soil improvement, carbon sequestration and slope stabilization.
